# Differential Binding and Neutralising Antibody Responses Across COVID-19 Severity in a Saudi Multicentre Cohort

**DOI:** 10.3390/v18070696

**Published:** 2026-06-24

**Authors:** Mariam M. AlEissa, Nada Saleh, Ahdab A. Alsaieedi, Raghad A. AlQurashi, Esraa A. Hawsa, Muath ben Shaded, Amer M. Alshehri, Eyad Y. Abu Sarhan, Osamah T. Khojah, Walid A. Nouh, Sami S. Almudarra, Khaled I. AlAbdulkareem, Ghada Garaween, Maha Alzayer, Yusra Alyafee, Monera Alrukhayes, Reema Alduaiji, Fahad A. Almsned, Abdullah M. Assiri

**Affiliations:** 1 College of Medicine, Alfaisal University, Riyadh 11533, Saudi Arabia; 2 Population Health Agency, Ministry of Health, Riyadh 11176, Saudi Arabia; ggaraween@alfaisal.edu; 3 Public Health Authority, Public Health Laboratory, Riyadh 11176, Saudi Arabia; 4 King Khaled Eye Specialist Hospital (KKESH) Research Center, Riyadh 11462, Saudi Arabia; 5 Computational Sciences Department at the Centre for Genomic Medicine (CGM), King Faisal Specialist Hospital and Research Center, Riyadh 11211, Saudi Arabia; 6 College of Medicine, King Saud Bin Abdulaziz University for Health Sciences (KSAU-HS), Riyadh 11481, Saudi Arabia; salehn@ksau-hs.edu.sa (N.S.);; 7 King Abdullah International Medical Research Centre (KAIMRC), Riyadh 11426, Saudi Arabia; 8 Ministry of National Guard Health Affairs (MNGHA), Riyadh 11426, Saudi Arabia; 9 Department of Medical Laboratory Sciences, Faculty of Applied Medical Sciences, King Abdulaziz University, Jeddah 21589, Saudi Arabia; aalsaieedi@kau.edu.sa; 10 King Fahd Medical Research Center, King Abdulaziz University, Jeddah 21589, Saudi Arabia; 11 Medical Diagnostic Laboratories, Dr. Sulaiman Alhabib Medical Group, Riyadh 12511, Saudi Arabiaasd@ksu.edu.sa (O.T.K.);; 12 Pathology Department, College of Medicine, King Saud University, Riyadh 12372, Saudi Arabia; 13 Gulf Centre for Disease Prevention and Control, Gulf Health Council, Riyadh 11481, Saudi Arabia; 14 Department of Primary Health Care, Ministry of Health, Riyadh 11176, Saudi Arabia; 15 Department of Medical Laboratory Sciences, Faculty of Allied Health Sciences, Health Sciences Center (HSC), Kuwait University, Jabriya 90805, Kuwait; 16 John Hopkins Aramco Healthcare, Dhahran 31311, Saudi Arabia; 17 Research Center, King Fahad Specialist Hospital in Dammam (KFSH-D), Dammam 32253, Saudi Arabia; falmsned@moh.gov.sa; 18 Population Health Management, Eastern Health Cluster, Dammam 32253, Saudi Arabia; 19 School of Systems Biology, George Mason University, Fairfax, VA 22030, USA

**Keywords:** COVID-19 severity, SARS-CoV-2, humoral immune response, neutralising antibodies, IgG, IgM, immunosenescence, Saudi Arabia, pandemic

## Abstract

Background: Humoral immune responses to SARS-CoV-2 are well documented, yet the immunopathogenic mechanisms distinguishing severe from critical disease remain incompletely defined, particularly in Middle Eastern populations. We investigated antibody responses across levels of clinical severity in a Saudi Arabian cohort. Methods: In this multicentre study, we analysed 406 participants stratified into five clinical groups: controls, asymptomatic, mild, severe, and critically ill requiring intensive care unit (ICU) admission. SARS-CoV-2-specific IgG and IgM levels were quantified alongside surrogate ACE2-RBD neutralisation activity. Associations between humoral markers, demographic factors, comorbidities, and disease severity were assessed. Results: SARS-CoV-2-specific IgG and IgM levels differed significantly across disease severity groups (*p* < 0.001), with higher levels observed in groups with greater clinical severity. No significant difference in IgG or IgM levels was observed between the severe and ICU groups (IgG *p* = 0.384; IgM *p* = 0.768). While binding antibody levels were associated with severity, surrogate ACE2-RBD neutralising activity did not differ significantly across groups (*p* = 0.209). Increasing age (χ^2^ = 44.5) and the presence of comorbidities (χ^2^ = 31.9) were associated with more severe clinical categories, whereas sex was not. Conclusions: These findings suggest that antibody levels provide useful information about exposure and immune activation, but antibody quantity alone does not fully explain the transition from severe to critical disease. The results support interpreting serological measures alongside clinical factors such as age and chronic illness.

## 1. Introduction

Since its emergence in December 2019, severe acute respiratory syndrome coronavirus 2 (SARS-CoV-2) has triggered a global pandemic of unprecedented scale, causing over 676 million confirmed cases and nearly 7 million deaths worldwide [1,2,3]. Even in the current endemic phase, the virus continues to evolve, posing ongoing challenges to global health systems. While approximately 80% of infections result in asymptomatic or mild disease, 15% of patients progress to severe illness requiring hospitalisation, and 5% develop critical respiratory failure necessitating intensive care [4]. Although advanced age and comorbidities are well-established predictors of mortality, severe disease frequently occurs in younger, otherwise healthy individuals, implicating additional host-specific immunological factors in disease pathogenesis [5].

Humoral immunity plays a central role in viral clearance, with SARS-CoV-2-specific IgM seroconverting within 3–5 days and IgG emerging by days 7–14 to drive long-term neutralisation [6]. However, the relationship between binding antibody levels and clinical outcome is complex. Several studies have shown that patients with more severe COVID-19 may have higher antibody titres than those with mild disease, challenging the assumption that higher antibody levels necessarily reflect better clinical protection [7]. This dissociation suggests that antibody quantity alone may not fully capture the quality, timing, or coordination of the humoral response during severe infection [8].

Furthermore, the majority of the immunological data originates from Western or East Asian cohorts, which has left the Middle East significantly underrepresented in the global literature. This gap is particularly critical given the region’s unique epidemiological history with MERS-CoV, which has circulated in Saudi Arabia since 2012 [6,9]. Exposure to related betacoronaviruses may have shaped population-level immunity in ways that distinctively influence SARS-CoV-2 responses, which makes this demographic essential for a comprehensive understanding of coronavirus immunopathogenesis [9,10].

In this multicentre study, we characterised SARS-CoV-2-specific IgM, IgG, and surrogate ACE2-RBD neutralising antibody activity across the clinical spectrum of COVID-19 in a Saudi cohort. We examined whether binding antibody levels and measured neutralising activity would diverge in severe versus critical disease and whether these serological markers contributed to severity classification alongside established host factors such as age, sex, and comorbidity. Rather than assuming that higher antibody levels necessarily indicate protection, this study was designed to describe how quantitative and functional antibody measures relate to clinical severity in this underrepresented population.

## 2. Materials and Methods

### 2.1. Ethical Approval

The study protocol adhered to the principles of the Declaration of Helsinki and was approved by the Central Institutional Review Board at the Ministry of Health, Saudi Arabia (Log No: 20-108M; Registration: H-01-R-009) on 14 June 2020. Written informed consent was obtained from all participants or their legal guardians prior to study enrolment.

### 2.2. Sample Recruitment

#### Study Design and Participant Recruitment

This multicentre observational study enrolled participants through a collaborative network coordinated by the Saudi Ministry of Health (MOH) and the Public Health Authority (PHA) across multiple clinical sites in Saudi Arabia between 30 October 2020 and 30 October 2021. The study population included males and females aged 12–100 years with SARS-CoV-2 infection confirmed by reverse transcription–polymerase chain reaction (RT-PCR) [11]. To ensure serological specificity, participants with evidence of respiratory viral co-infections other than SARS-CoV-2 were excluded.

A total of 406 participants were recruited based on consecutive eligibility during the study period and stratified across the clinical spectrum of COVID-19: asymptomatic infection (n = 110), mild disease (n = 179), severe disease requiring hospitalisation (n = 63), and critical disease requiring intensive care unit (ICU) admission (n = 34). Disease severity classification followed the World Health Organization (WHO) guidance (WHO/2019-nCoV/Clinical/2020.5) [12], with categorisation assigned based on documented clinical status during the acute episode. Demographic data and comorbidities (e.g., diabetes, hypertension) were recorded via self-report and verified against medical records where available.

A control group (n = 20) was recruited among individuals with documented close-contact exposure to confirmed COVID-19 cases for more than three days who tested negative by RT-PCR and reported no acute illness at enrolment. Control sera were collected at least 14 days post-exposure to allow for potential seroconversion detection, which provided a robust contemporaneous comparator group.

Where available, serum samples were collected after documented infection or exposure and processed for serological testing. Exact timing from symptom onset, diagnosis, hospital admission, or ICU admission was not consistently available across sites. Therefore, antibody comparisons should be interpreted as cross-sectional group comparisons rather than longitudinal kinetic measurements.

Vaccination status and infecting viral variant were not systematically captured in the study database. Because recruitment extended into 2021, some participants may have received COVID-19 vaccination before sampling. Similarly, variant-specific viral sequencing was not available. The serological assays used manufacturer-supplied SARS-CoV-2 antigens rather than participant-derived viral isolates; therefore, the results reflect binding and ACE2-RBD inhibition measured against the assay antigens and should not be interpreted as variant-specific neutralisation.

### 2.3. Immunological Assays

Venous blood samples were collected, centrifuged to isolate serum, and stored at −80 °C until analysis. Quantitative SARS-CoV-2-specific IgG antibodies targeting the receptor-binding domain (RBD) of the S1 subunit of the spike protein were measured using the Abbott SARS-CoV-2 IgG II Quant chemiluminescent microparticle immunoassay (CMIA; Abbott Laboratories, Abbott Park, IL, USA) on the ARCHITECT i System according to the manufacturer’s protocols. The manufacturer-defined seropositivity cutoff was ≥50.0 AU/mL (manufacturer-reported sensitivity: 98.8%; specificity: 99.6%).

For the detection of SARS-CoV-2-specific IgM, the Vircell COVID-19 ELISA IgM kit (Vircell S.L., Granada, Spain) was employed, targeting both the nucleocapsid (N) and spike (S) proteins. IgM results were interpreted as positive, equivocal, or negative based on the manufacturer-defined optical density (OD) index. Because the IgG and IgM assays measure different antibody targets and use different laboratory readouts, the results were analysed separately as binding antibody responses rather than combined into one overall antibody response score.

#### Surrogate Neutralising Antibody Assessment

Surrogate neutralising antibody activity was assessed in a subset of serum samples (n = 86) across severity groups, contingent on sample volume availability. We employed the SARS-CoV-2 Neutralizing Ab ELISA Kit (BMS2326; Invitrogen™, Thermo Fisher Scientific, Waltham, MA, USA), a surrogate ACE2-RBD competitive inhibition enzyme-linked immunosorbent assay that measures the ability of serum antibodies to block the interaction between the viral spike RBD and the host ACE2 receptor. Briefly, serum samples were diluted 1:50 and incubated with an RBD-coated microplate, followed by the addition of biotinylated ACE2, which allowed neutralising antibodies to compete for RBD binding sites. The percentage of neutralisation (inhibition) was calculated as:

Neutralisation (%) = [1 − (OD450 of sample/OD450 of negative control)] × 100

Results represent the mean inhibition of duplicate wells. A cutoff of ≥20% inhibition was defined as positive for neutralising activity, a threshold previously validated to correlate with live virus microneutralisation titres.

**Figure 1:**
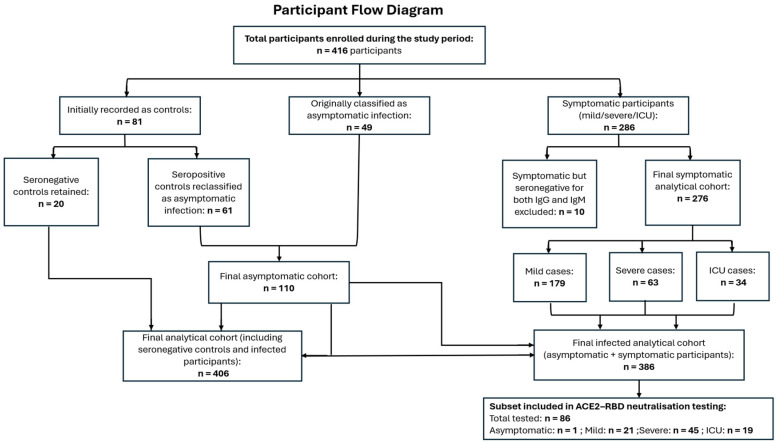
Participant flow diagram showing recruitment, exclusions, reclassification of seropositive controls as asymptomatic infections, exclusion of symptomatic seronegative participants, final analytical severity groups, and the subset included in ACE2-RBD neutralisation testing.

A data-quality and reclassification step was conducted before the final analysis. Participants initially recorded as uninfected controls but testing seropositive for SARS-CoV-2 IgG/IgM (n = 61) were reclassified as asymptomatic infections, as serology indicated prior exposure in the absence of reported symptoms. Conversely, symptomatic participants (mild/severe/ICU) who tested seronegative for both IgG and IgM (n = 10) were excluded from the final serological analysis to minimise potential misclassification related to PCR/serology discordance or timing of sampling. Figure 1 shows how the final analytical cohort was derived, including participant recruitment, exclusions, reclassification, and the subset selected for neutralisation testing.

### 2.4. Statistical Analysis

Statistical analyses were performed using IBM SPSS Statistics v30.0 (IBM Corp., Armonk, NY, USA). Continuous variables (antibody titres) were assessed for normality using the Shapiro–Wilk test; given their non-normal distribution, data are presented as medians (interquartile range, IQR) and compared across severity groups using the Kruskal–Wallis test followed by Dunn’s post hoc test with Bonferroni correction. Categorical variables were expressed as frequencies and percentages, with associations assessed using Pearson’s Chi-square test. To identify factors associated with clinical severity, a multinomial logistic regression model was constructed with disease severity (Control [reference], Asymptomatic, Mild, Severe, ICU) as the dependent variable and age, sex, presence of comorbidities, and antibody status (IgG/IgM) as covariates. Model fit was assessed using the Likelihood Ratio Chi-square test and Pseudo R-squared metrics (Cox & Snell, Nagelkerke). Odds ratios (ORs) with 95% confidence intervals (CIs) were calculated for each severity category. 

## 3. Results

After reclassification and exclusions, the final analytical cohort comprised 406 participants: 20 exposed RT-PCR-negative controls, 110 asymptomatic infections, 179 mild cases, 63 severe cases requiring hospitalisation, and 34 critical cases requiring ICU admission (Figure 1). The cohort included 236 males (58.1%) and 170 females (41.9%). Nearly half the cohort (48.3%) was aged 31–60 years, while paediatric participants (<18 years) constituted a minority (2.2%). Approximately one-quarter of the participants (24.4%, n = 99) reported at least one underlying comorbidity, with diabetes mellitus (15.0%) and hypertension (13.8%) being the most prevalent conditions (Table 1).

The multivariable multinomial logistic regression model demonstrated robust predictive validity, significantly outperforming the null model [χ^2^(20) = 257.83, *p* < 0.001]. The selected predictors accounted for a substantial proportion of the variance in disease severity (Nagelkerke R^2^ = 0.532; Cox & Snell R^2^ = 0.498). The model achieved an overall classification accuracy of 50.5%, which supports its usefulness for describing associations across the five severity categories.

### 3.1. Association Between Binding Antibody Levels and Disease Severity

**Figure 2:**
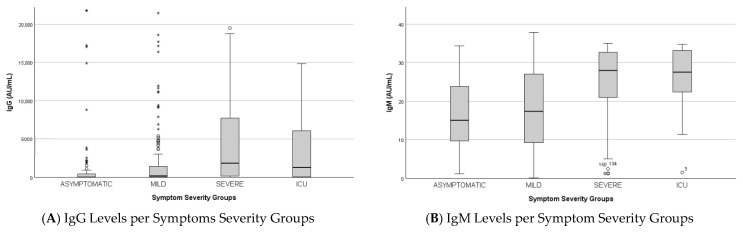
IgG and IgM levels across symptom severity groups. The control group was excluded because all participants had negative IgG and IgM results. (*) indicate outliers. Within-group variability reflects differences in infection history among participants as shown in Table S1.

Quantitative analysis revealed an association between SARS-CoV-2 antibody levels and disease severity. Both IgG [χ^2^(4) = 50.64, *p* < 0.001] and IgM [χ^2^(4) = 88.78, *p* < 0.001] levels were significantly elevated in the infected cohorts relative to the controls, with higher antibody levels observed in groups with greater clinical severity (Figure 2). A Kruskal–Wallis test confirmed significant heterogeneity in the median antibody levels across the clinical spectrum for both IgG [χ^2^(3) = 43.99, *p* < 0.001] and IgM [χ^2^(3) = 52.89, *p* < 0.001]. Post hoc pairwise comparisons (Dunn’s test) showed that asymptomatic individuals had significantly lower antibody levels than those with symptomatic disease (*p* < 0.001), while patients with severe disease had significantly higher IgG and IgM levels compared to mild cases (*p* < 0.001). Notably, no significant difference in antibody levels was observed between the severe and ICU groups, which suggests that the increase in binding antibody levels did not continue across the transition from severe to critical illness (Supplementary Tables S4 and S5).

### 3.2. Sex Distribution Across Disease Severity Groups

**Figure 3:**
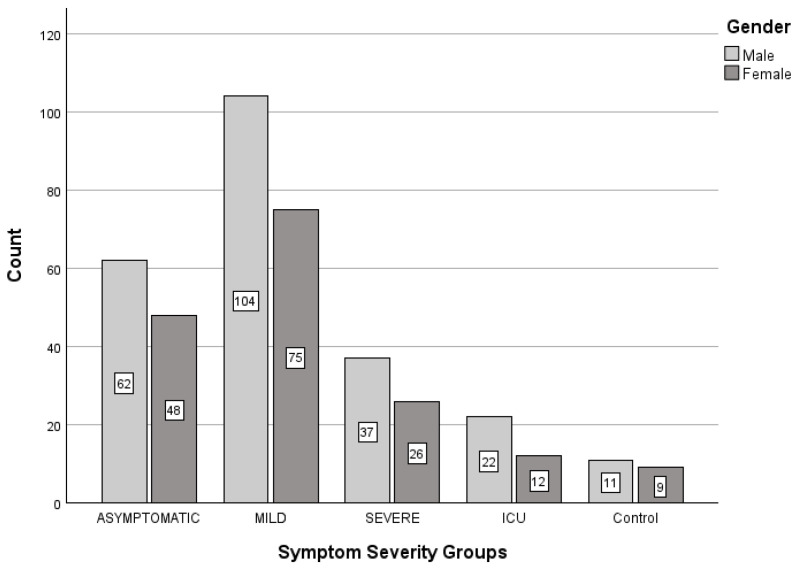
Sex distribution across COVID-19 severity groups.

**Figure 4:**
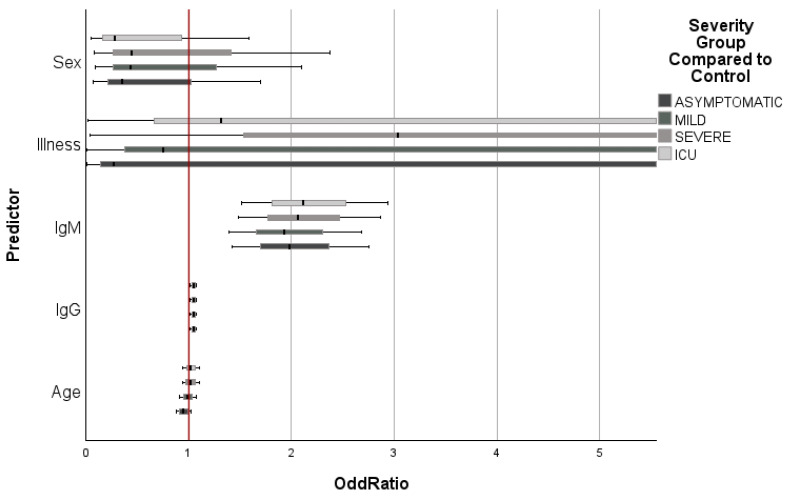
Forest plot of adjusted odds ratios and 95% confidence intervals from multinomial logistic regression examining associations between age, sex, chronic illness, IgG status, IgM status, and COVID-19 severity category. The model was adjusted for all listed variables. Odds ratios should be interpreted as associations with severity category, not as causal effects.

Sex was not significantly associated with disease severity in the multinomial logistic regression model (χ^2^(4) = 3.31, *p* = 0.506), which indicates that the male and female participants did not differ significantly in their odds of belonging to the severity groups relative to the control group (Figure 3, Supplementary Tables S2 and S3). To improve interpretability, the principal adjusted associations are summarised in a forest plot, Figure 4. Given the cross-sectional design and the absence of consistently available timing and vaccination data, regression findings were interpreted as associations rather than causal predictors of disease progression. A two-tailed *p*-value <0.05 was considered statistically significant.

### 3.3. Demographic and Clinical Factors Associated with Disease Severity

Demographic and clinical factors played a variable role in predicting disease severity. Increasing age was associated with disease severity [χ^2^(4) = 44.53, *p* < 0.001], with older participants significantly more likely to be classified into the severe or critical disease categories. Similarly, the presence of underlying chronic health conditions was also associated with more severe clinical categories [χ^2^(4) = 31.96, *p* < 0.001], where participants with at least one comorbidity demonstrated significantly higher odds of developing severe symptoms or requiring ICU admission compared to healthy individuals.

**Figure 5:**
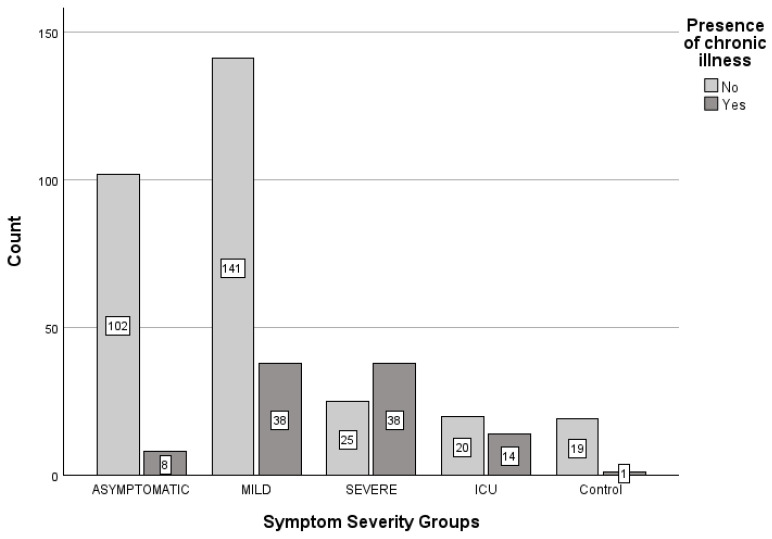
Presence of chronic illness across COVID-19 severity groups.

Consistent with the regression findings, having one or more chronic illnesses was strongly associated with higher disease severity (χ^2^(4) = 31.96, *p* < 0.001), which indicates that participants with comorbidities had higher odds of more severe COVID-19 outcomes (Figure 5, Supplementary Tables S2 and S3).

### 3.4. Surrogate ACE2-RBD Neutralising Antibody Activity Across Severity Groups

**Figure 6:**
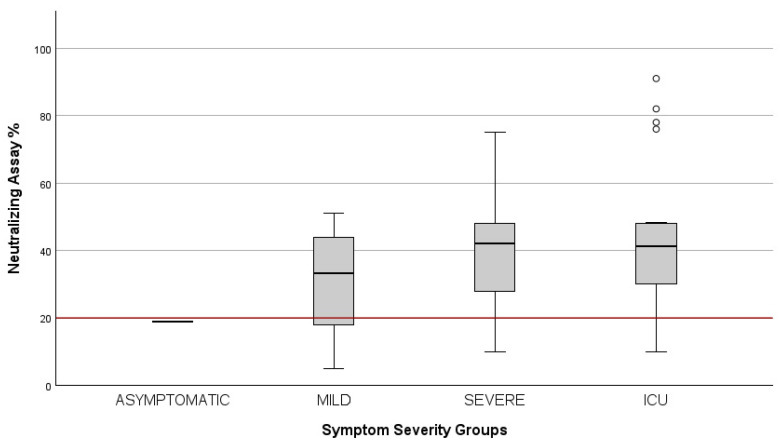
Surrogate ACE2-RBD neutralisation activity. Assay result distributed by COVID-19 severity group in neutralisation subset (n = 86). Percentage inhibition was measured using a competitive ACE2-RBD ELISA, with ≥20% inhibition indicating detectable neutralising activity according to the manufacturer’s cutoff. Group sizes were asymptomatic n = 1, mild n = 21, severe n = 45, and ICU n = 19. The small and uneven group sizes limit between-group interpretation.

Surrogate ACE2-RBD neutralising antibody activity was assessed in a subset of 86 participants with sufficient residual serum: asymptomatic (n = 1), mild (n = 21), severe (n = 45), and ICU (n = 19). A Kruskal–Wallis analysis revealed no statistically significant differences in percentage inhibition across clinical severity groups [χ^2^(3) = 4.54, *p* = 0.209] (Figure 6). Because the neutralisation subset was small and unevenly distributed, particularly for the asymptomatic group, this finding should be interpreted as an absence of detected statistical difference rather than evidence that neutralising protection was equivalent across severity categories.

## 4. Discussion

In this multicentre Saudi cohort, we examined SARS-CoV-2-specific binding IgG, binding IgM and surrogate ACE2-RBD neutralising activity across COVID-19 severity groups. Binding IgG and IgM levels were higher across the clinical spectrum, particularly when comparing asymptomatic or mild infection with severe disease, but they did not clearly separate severe cases from ICU cases. In the smaller neutralisation subset, ACE2-RBD inhibition also did not differ significantly between severity groups. Age and chronic illness showed the strongest associations with the more severe clinical categories, whereas sex was not independently associated with severity. Taken together, these findings suggest that antibody levels alone do not fully explain the immunological differences between severe and critical COVID-19.

In this study, both SARS-CoV-2-specific IgM and IgG levels rose in parallel with clinical severity, with higher antibody levels consistently associated with increased odds of belonging to a COVID-19-positive severity category compared to the controls. This pattern aligns with prior clinical cohorts showing that humoral responses typically emerge within the first 2–3 weeks after symptom onset and that higher antibody titres can be observed in patients with more severe clinical phenotypes, reflecting greater antigenic burden and/or more intense immune activation [13,14]. Comparatively, our data indicate that the rise was most pronounced in severe (non-ICU) disease versus mild illness, and the ICU group did not demonstrate the same clear distinction from mild cases. The absence of a direct association between antibody quantity and clinical category has been documented in various studies and may reflect sampling timepoints, class-switching kinetics, and the complexity of humoral responses in severe illness. For example, longitudinal studies show that ICU patients may exhibit dysregulated or poorly coordinated humoral responses, including less efficient switching to spike-specific IgG over time despite robust nucleocapsid responses [15]. Furthermore, among severely ill ICU patients, delayed or absent early antibody responses have been linked to poor clinical outcomes in some studies, which suggests that critical illness may encompass both hyperinflammatory phenotypes with robust antibody production and immune-exhausted phenotypes with impaired humoral responses [16].

We identified a correlation between elevated levels of IgM and disease progression, including admission to the ICU. Typically, IgM is regarded as an early biomarker of the initial stages of infection, reflecting recent antigen exposure and early humoral activation. Variability in IgM responses may be influenced by viral burden, the timing of sample collection, or host immune competence. Exaggerated or prolonged IgM responses are generally interpreted as markers of persistent antigenic exposure/stimulation and dysregulated immune activation rather than effective viral clearance [13,17]. Severe cases of COVID-19 have been shown to mount vigorous early humoral responses that may coexist with maladaptive inflammatory immune profiles, which supports the notion that elevated IgM responses are not necessarily protective [18]. Collectively, these observations are consistent with evidence that the most severe forms of COVID-19 are characterised by intense but poorly coordinated humoral immune responses [19,20].

In this cohort, binding antibody levels generally increased with clinical severity, but this increase was not found when severe cases were compared with ICU cases. This pattern should not be taken as evidence of a separate nonlinear mechanism. Rather, it suggests that the relationship between antibody levels and clinical severity is not uniform across all disease categories. This may reflect several factors, including differences in sampling time, antigenic burden, class-switching kinetics, inflammatory status, and the clinical heterogeneity of critical illness. Overall, these findings should be interpreted as descriptive evidence that binding antibody levels alone have a limited ability to distinguish severe from critical disease.

Antibody levels increased significantly from mild to severe disease but did not show a further increase in ICU patients. This pattern suggests that binding antibody levels alone may have a limited ability to distinguish severe from critical illness rather than indicating a nonlinear mechanism. A similar plateauing or attenuation of antibody responses has been reported in critically ill COVID-19 patients and may reflect disrupted germinal centre reactions, altered T follicular helper cell support, and late-stage immune dysfunction or differences in sampling time rather than insufficient antigen exposure alone [20,21,22]. By contrast, although IgG levels were elevated in infected individuals compared to uninfected controls, SARS-CoV-2-specific IgG levels were not associated with disease severity. This is consistent with previous observations that total binding IgG titres do not reliably distinguish mild from severe cases of COVID-19 [17,19]. IgG responses typically develop later than IgM responses and persist beyond the acute phase of infection, serving primarily as indicators of prior exposure and immunological memory rather than acute disease trajectory. Furthermore, in severe cases, many patients have high IgG levels, which reinforces the concept that elevated antibody titres alone do not necessarily equate to effective immune control [18].

Importantly, surrogate ACE2-RBD inhibitory activity did not differ significantly between severity groups in the analysed subset. This finding should be interpreted with care, as the neutralisation analysis was performed in a smaller subset than that used for the binding antibody analyses, with uneven group sizes and only one asymptomatic sample. Neutralising antibodies are recognised as important correlates of protection against SARS-CoV-2 infection; however, a single threshold that clearly distinguishes between mild, severe, and critical disease during primary infection has not been established. Therefore, our data are best interpreted as showing that measurable ACE2-RBD inhibitory activity was present across severity groups rather than indicating that protective immunity was equivalent between groups. This distinction is important because ACE2-RBD competitive inhibition assays provide a practical surrogate measure of neutralising activity, but they mainly capture antibodies that block RBD-ACE2 interaction and do not fully reflect the broader functional activity measured by live-virus or pseudovirus neutralisation assays. Longitudinal studies using live-virus or pseudovirus neutralisation methods would be needed to better clarify how neutralising activity relates to clinical progression [23,24].

It is also important to distinguish the surrogate ACE2-RBD inhibition assay used in this study from live-virus plaque reduction neutralisation and pseudovirus neutralisation assays. The competitive ELISA provides a practical measure of antibodies that interfere with the interaction between spike RBD and ACE2, but it does not capture the full range of functional antibody responses [23]. For example, antibodies directed against non-RBD spike regions, including the N-terminal domain and S2 region, may contribute to epitope breadth [25], while Fc-mediated antibody functions may also contribute to the overall antiviral response [26]. This distinction is relevant because infection- and vaccine-induced antibody repertoires can differ in epitope breadth and functional profile. Therefore, the neutralisation findings in this study should be interpreted as RBD-directed ACE2-blocking activity rather than as a complete measure of antiviral neutralising capacity.

Older age was a strong independent predictor of disease severity in this cohort, consistent with findings from large international studies identifying age as a major risk factor for adverse COVID-19 outcomes [14,27]. In addition, pre-existing chronic diseases were more frequently observed among patients with greater clinical severity. From a biological perspective, age-associated immune alterations provide a plausible framework for these observations. Immune senescence and inflammaging have been linked to impaired early antiviral responses, reduced adaptive immune flexibility, and a heightened tendency towards dysregulated inflammatory responses [28,29]. Together, these age- and comorbidity-related immune changes may increase susceptibility to severe disease by limiting effective viral control while promoting immune-mediated pathology.

The presence of chronic illness, particularly metabolic and cardiovascular conditions, was also associated with greater disease severity in this cohort. Such comorbidities have been widely reported as risk factors for adverse COVID-19 outcomes and are known to influence immune homeostasis, endothelial function, and inflammatory regulation, thereby increasing vulnerability to severe viral infection [27,30]. In the Saudi Arabian context, where the prevalence of non-communicable diseases is high, these findings carry important public health relevance and warrant the need for targeted risk stratification and early clinical intervention strategies [31].

By contrast, sex was not independently associated with disease severity in this analysis. Although sex-based differences in COVID-19 outcomes have been reported in some populations [27,30], our findings suggest that when immune markers, age, and comorbidities are considered simultaneously, sex alone does not substantially influence disease severity. This observation underscores the value of multivariable, immune-centric analytical approaches when assessing host susceptibility to severe infection.

This study has several strengths, including its multicentre design, inclusion of patients spanning the full clinical spectrum of COVID-19, and careful reclassification of seropositive asymptomatic individuals, which helped reduce misclassification bias [17]. Nevertheless, several limitations should be acknowledged. The cross-sectional design limits assessment of longitudinal immune dynamics and causal inference. In addition, the timing of sample collection in relation to symptom onset, diagnosis, hospitalisation, or ICU admission was not consistently available, which may have influenced antibody levels across severity groups. Key components of antiviral immunity, such as cell-mediated immune responses, virus-specific T-cell activity, and memory B-cell responses, were not evaluated, despite their established roles in viral control and long-term protection [19]. Vaccination status and information on circulating viral variants were also unavailable and could not be included in the analysis. This study did not directly quantify non-neutralising antibodies or calculate the proportion of binding antibodies that lacked neutralising activity. Binding IgG, binding IgM, and ACE2-RBD inhibition were measured using different assay platforms, antigenic targets, and readouts; therefore, a reliable ratio of neutralising to non-neutralising antibodies could not be derived from these data.

In conclusion, this Saudi multicentre cohort provides evidence that SARS-CoV-2 binding IgG and IgM levels increase across the clinical severity spectrum, particularly from asymptomatic or mild infection to severe disease, but do not further distinguish severe from ICU disease. Surrogate ACE2-RBD inhibitory activity was detectable across severity groups but did not differ significantly in the analysed subset, a finding that should be considered in the context of the smaller sample size and assay type. Age and chronic illness showed the clearest associations with more severe clinical categories. Together, these findings highlight the value of interpreting serological measures alongside clinical factors and suggest that antibody quantity alone is insufficient to explain the transition from severe to critical illness.

## Figures and Tables

**Table 1:** Descriptive characteristics of participants in this study.

	Female (n = 170, 41.9%)	Male (n = 236, 58.1%)	Total (n = 406, 100%)
Age group			
Under 18	3 (0.7%)	6 (1.5%)	9 (2.2%)
18–30	38 (9.4%)	42 (10.3%)	80 (19.7%)
31–45	46 (11.3%)	60 (14.8%)	106 (26.1%)
46–60	39 (9.6%)	51 (12.6%)	90 (22.2%)
61 and above	35 (8.6%)	54 (13.3%)	89 (21.9%)
Unknown	9 (2.2%)	23 (5.7%)	32 (7.9%)
Does the participant have a chronic illness?			
Yes	36 (8.9%)	63 (15.5%)	99 (24.4%)
No	134 (33.0%)	173 (42.6%)	307 (75.6%)
Type of chronic illness			
Dementia	1 (0.2%)	1 (0.2%)	2 (0.5%)
Blood disorders	0 (0.0%)	1 (0.2%)	1 (0.2%)
Diabetes	24 (5.9%)	37 (9.1%)	61 (15.0%)
Hypertension	23 (5.7%)	33 (8.1%)	56 (13.8%)
Cholesterol	1 (0.2%)	0 (0.0%)	1 (0.2%)
Stroke	0 (0.0%)	2 (0.5%)	2 (0.5%)
Cancer	0 (0.0%)	1 (0.2%)	1 (0.2%)
Renal disease	1 (0.2%)	6 (1.5%)	7 (1.7%)
Cardiac problems	3 (0.7%)	5 (1.2%)	8 (1.9%)
Rhesus disease	0 (0.0%)	0 (0.0%)	0 (0.0%)
Respiratory failure	1 (0.2%)	1 (0.2%)	2 (0.5%)
Asthma	2 (0.5%)	2 (0.5%)	4 (1.0%)

## Data Availability

Data is available upon request. Please contact Mariam AlEissa maaleissa@alfaisal.edu.

## Supplementary Materials

The following supporting information can be downloaded at: https://www.mdpi.com/article/10.3390/v18070696/s1, Table S1: Descriptive statistics of the five COVID-19 symptom-severity groups alongside their IgG and IgM results; Table S2: Chi-square test results for associations between predictors and symptom severity groups; Table S3: Multinomial Logistic Regression Odds Ratios for Classification Into COVID-19 Symptom Severity Groups Relative to the Control Group; Table S4: Kruskal–Wallis comparisons of IgG and IgM levels across symptom severity groups; Table S5: Post hoc (Dunn’s Test) pairwise comparisons of IgG and IgM levels across symptom severity groups.
