# Supplementary material for: Differential Binding and Neutralising Antibody Responses Across COVID-19 Severity in a Saudi Multicentre Cohort
Source: Viruses. 2026 Jun 24;18(7):696. doi: 10.3390/v18070696 (PMC13431541; doi:10.3390/v18070696)
Supplement: Supplementary file 1 [file viruses-18-00696-s001.zip › viruses-4347570-supplementary.pdf]

Supplementary tables

Table S1: Descriptive statistics of the five COVID-19 symptom-severity groups alongside their IgG and IgM results

Severity Classification	IgG Result		IgM Result		Total
	Positive	Negative	Positive	Negative	
Control	0 (0.0%)	20 (4.9%)	0 (0.0%)	20 (4.9%)	20 (4.9%)
Asymptomatic	49 (12.1%)	61 (15.0%)	95 (23.4%)	15 (3.7%)	110 (27.1%)
Mild	131 (32.3%)	48 (11.8%)	149 (36.7%)	30 (7.4%)	179 (44.1%)
Severe	52 (13.8%)	11 (2.7%)	58 (14.3%)	5 (1.2%)	63 (15.5%)
ICU	26 (6.4%)	8 (2.0%)	33 (8.1%)	1 (0.2%)	34 (8.4%)

Table S2: Chi-square test results for associations between predictors and symptom severity groups

**Predictor**	**Chi-Square**	**df**	**p-value**
IgG	50.6	4	**< 0.001***
IgM	88.7	4	**< 0.001***
Gender	3.3	4	0.506
Age	44.5	4	**< 0.001***
Presence of Chronic Disease	31.9	4	**< 0.001***

* $p < 0.05$

Table S3: Multinomial Logistic Regression Odds Ratios for Classification Into COVID-19 Symptom Severity Groups Relative to the Control Group

Symptom severity group	Predictor	Value	Reference	OR	95% CI	p-value
**Asymptomatic**	IgG	X + 1	X	1.045	1.01, 1.08	**0.004***
	IgM	X + 1	X	1.979	1.42, 2.75	**< 0.001***
	Gender	F	M	0.347	0.07, 1.69	0.191
	Age	X + 1	X	0.946	0.87, 1.03	0.178
	Presence of Chronic Disease	with chronic disease	without chronic disease	0.268	0.00, 20.17	0.550
**Mild**	IgG	X + 1	X	1.045	1.01, 1.08	**0.004***
	IgM	X + 1	X	1.932	1.39, 2.68	**< 0.001***
	Gender	F	M	0.437	0.09, 2.1	0.301
	Age	X + 1	X	0.988	0.91, 1.07	0.762
	Presence of Chronic Disease	with chronic disease	without chronic disease	0.748	0.01, 53.4	0.894
**Severe**	IgG	X + 1	X	1.045	1.01, 1.08	**0.004***
	IgM	X + 1	X	2.059	1.48, 2.87	**< 0.001***
	Gender	F	M	0.444	0.08, 2.38	0.343
	Age	X + 1	X	1.015	0.94, 1.1	0.728
	Presence of Chronic Disease	with chronic disease	without chronic disease	3.036	0.04, 224.77	0.613
**ICU**	IgG	X + 1	X	1.045	1.01, 1.08	**0.004***
	IgM	X + 1	X	2.111	1.51, 2.94	**< 0.001***
	Gender	F	M	0.274	0.05, 1.58	0.148
	Age	X + 1	X	1.017	0.94, 1.1	0.695
	Presence of Chronic Disease	with chronic disease	without chronic disease	1.315	0.02, 100.26	0.901

* $p < 0.05$

Table S4: Kruskal–Wallis comparisons of IgG and IgM levels across symptom severity groups

Variable	Chi-Square	df	p-value

IgG Result	43.9	3	**< 0.001***
IgM Result	52.9	3	**< 0.001***

* $p < 0.05$

Table S5: Post hoc (Dunn's Test) pairwise comparisons of IgG and IgM levels across symptom severity groups

Variable	Pairwise comparison	p-value
IgG Result	Asymptomatic – Mild	**< 0.001***
	Asymptomatic – ICU	**< 0.001***
	Asymptomatic – Severe	**< 0.001***
	Mild – ICU	0.108
	Mild – Severe	**< 0.001***
	ICU – Severe	0.384
IgM Result	Asymptomatic – Mild	0.425
	Asymptomatic – ICU	**< 0.001***
	Asymptomatic – Severe	**< 0.001***
	Mild – ICU	**< 0.001***
	Mild – Severe	**< 0.001***
	ICU – Severe	0.768

* $p < 0.05$
